# Real-world efficacy and safety of switching to bictegravir/emtricitabine/tenofovir alafenamide in older people living with HIV

**DOI:** 10.1097/MD.0000000000027330

**Published:** 2021-09-24

**Authors:** Charlotte-Paige Rolle, Vu Nguyen, Kiran Patel, Dan Cruz, Edwin DeJesus, Federico Hinestrosa

**Affiliations:** a Orlando Immunology Center, Orlando, FL; b Department of Global Health, Emory University Rollins School of Public Health, Atlanta, GA; c University of Central Florida College of Medicine, Orlando, FL; d Gilead Sciences, Foster City, CA.

**Keywords:** bictegravir, drug–drug interactions, efficacy, older adults, real-world, safety

## Abstract

Approximately 50% of people living with HIV (PLWH) in the United States are ≥50 years old. Clinical trials of bictegravir/emtricitabine/tenofovir alafenamide (B/F/TAF) demonstrated potent efficacy and favorable safety in older PLWH; however, real-world data would be useful to validate these results.

Retrospective cohort study.

We evaluated records from PLWH aged ≥50 years at the Orlando Immunology Center who were switched to B/F/TAF between February 2018 and August 2019. Eligible patients had baseline HIV-1 RNA <50 copies/mL and 48 weeks of follow-up data. The primary endpoint was maintenance of HIV-1 RNA <50 copies/mL at Week 48. The impact of switching to B/F/TAF on drug–drug interactions (DDIs) and safety parameters were also assessed.

Three-hundred and fifty patients met inclusion criteria, median age was 57 years, 20% were women, and 43% were non-White. Fifty-five percent of patients switched from integrase inhibitor-based regimens; the most common reason for switch was simplification. At Week 48, 330 (94%) patients maintained an HIV-1 RNA <50 copies/mL and 20 (6%) had an HIV-1 RNA between 50 and 400 copies/mL. One-hundred and forty potential DDIs were identified in 121 (35%) patients taking a boosting agent or rilpivirine at baseline that were resolved after switching to B/F/TAF. Treatment-related adverse events occurred in 51 (15%) patients (all Grade 1–2) and led to 8 discontinuations.

In this real-world cohort, switching to B/F/TAF was associated with maintenance of virologic control, and avoidance of DDIs in a large proportion of patients. These data support use of B/F/TAF as a treatment option in older PLWH.

## 1 Introduction

Bictegravir/emtricitabine/tenofovir alafenamide (B/F/TAF) is approved for the treatment of HIV-1 infection in treatment-naïve and -experienced adults who are stable on their current antiretrovirals (ARVs).^[1]^ Switch studies of B/F/TAF suggested several benefits of this single-tablet regimen, which included fewer drug–drug interactions (DDIs), a low pill burden and improved metabolic parameters when compared with boosted protease inhibitor based regimens.^[2]^ These characteristics make B/F/TAF attractive for lifelong ART treatment, especially in older adults who are more prone to polypharmacy and metabolic complications. In 2018, surveillance data revealed that over half of people living with HIV (PLWH) in the United States are aged ≥50 years and 17% of new HIV diagnoses occurred among this group.^[3]^ Older PLWH are at increased risk of cardiovascular disease, impaired cognition, and accelerated kidney disease and osteoporosis, and some of this excess risk maybe related to long-term ART exposure.^[4]^ Therefore, it is important that we continue to generate long-term data on the safety and efficacy of ARVs in this population.

In clinical studies, B/F/TAF was non-inferior to 3-drug dolutegravir (DTG)-containing regimens among 196 treatment-naïve adults aged ≥50 years through 144 weeks.^[5,6]^ Among 511 virologically suppressed, treatment-experienced adults aged ≥50 years, B/F/TAF was non-inferior to 3-drug boosted PI- and DTG-containing regimens though 48 weeks, and virologic suppression was maintained in 99% to 100% of participants continuing B/F/TAF through 144 weeks.^[7,8]^ In a secondary analysis of patient-reported outcomes, B/F/TAF was associated with fewer bothersome symptoms compared with participants on dolutegravir/abacavir/lamivudine (DTG/ABC/3TC).^[9]^ Treatment-naïve participants receiving B/F/TAF reported less fatigue, nausea/vomiting, dizziness/light-headedness, and insomnia, whereas virologically suppressed participants receiving B/F/TAF reported less nausea/vomiting, sadness/depression, nervousness/anxiety, and insomnia compared with those on DTG/ABC/3TC.^[9]^ These symptoms are prevalent in older populations, and the use of ARVs that are associated with fewer bothersome symptoms in the elderly would be highly beneficial.

Clinical trials of B/F/TAF in adults aged ≥65 years demonstrate high efficacy and a favorable safety and tolerability profile in this population. Data from GS-US-380–4449 demonstrated that 94% of virologically suppressed adults aged ≥65 years who switched to B/F/TAF, the majority of whom were on elvitegravir/cobicistat/F/TAF at baseline, maintained virologic suppression at Week 72 despite a high proportion with baseline comorbidities requiring multiple concomitant medications.^[10]^ At Week 72, no participant had an HIV-1 RNA ≥50 copies/mL, and there were no cases of treatment-emergent resistance.^[10]^ B/F/TAF was well tolerated in this population with only 2% discontinuing due to drug-related adverse events (AEs); there were no discontinuations related to renal, bone, or hepatic AEs through Week 72.^[10]^ A Week 48 subgroup analysis demonstrated significant reductions in total cholesterol and triglycerides among those switched to B/F/TAF, with no significant changes in low density lipoprotein (LDL) cholesterol, high density lipoprotein (HDL) cholesterol, or total cholesterol to HDL ratio.^[11]^

A pooled analysis of 4 international trials that included data from GS-US-380–4449 revealed that among 140 virologically suppressed adults aged ≥65 years who switched to B/F/TAF, 92% maintained virologic suppression through Week 48.^[12]^ No participant developed virologic failure, and there was no treatment-emergent resistance observed. Only one participant discontinued B/F/TAF due to a drug-related AE, and there were no serious drug-related AEs. Modest improvements in fasting lipid parameters were observed, and only 4% initiated lipid-lowering therapy throughout the study.^[12]^

Real-world data from older PLWH who are switched to B/F/TAF are currently lacking. Results from the BICSTaR cohort which included a subset of participants from Germany, Canada, France, and the Netherlands demonstrated that 93% of 182 treatment-experienced patients aged ≥50 years who switched to B/F/TAF, the majority of whom were virologically suppressed at baseline, achieved an HIV-1 RNA <50 copies/mL at Month 12.^[13]^ Overall, 89% of treatment-experienced patients persisted on B/F/TAF at Month 12, and only 7.7% of discontinuations among this group were due to AEs. There were no discontinuations due to drug-related renal, hepatic, or bone events.^[13]^ An analysis of patient-reported outcomes data from this cohort revealed statistically higher treatment satisfaction scores among 209 treatment-experienced patients switched to B/F/TAF, 49% of whom were ≥50 years of age.^[14]^

These clinical trial and real-world data suggest that B/F/TAF has a favorable efficacy and safety profile in older PLWH; however, these studies were largely composed of White patients with few women, and only one study included data from patients in the United States (US). Real-world data from larger, more diverse cohorts of older PLWH in the US would be useful to validate these results.

## 2 Methods

This was a retrospective observational cohort study to describe the effectiveness, safety, and tolerability of switching to B/F/TAF in adults aged ≥50 years old through 48 weeks seen at the Orlando Immunology Center (OIC). The OIC is a private infectious disease practice located in downtown Orlando, Florida that provides HIV and primary care services to approximately 5700 PLWH. The center serves a diverse population of PLWH; 25% are women, 30% report Hispanic and or Latino ethnicity and 20% identify as black or African American. The OIC is staffed by 3 infectious disease physicians, 1 primary care physician, and 3 advanced practice registered nurses (APRNs) who have completed residencies in infectious disease. ARV management decisions are made by 1 of the 3 infectious disease physicians who supervise all patient visits where ARVs are initiated or switched.

Eligible patients included all PLWH who were switched to daily B/F/TAF as a complete ARV regimen between February 2018 and August 2019 and were aged ≥50 years old at the time of switch. Other inclusion criteria included the availability of 2 consecutive baseline HIV-1 RNA values <50 copies/mL (at least 3 months apart) in the year prior to switch, attendance at a minimum of 2 clinic visits in the year prior to switch, and attendance at ≥2 clinic visits during the study period with a minimum of 2 HIV-1 RNA measurements following switch to allow for an efficacy estimate. Key exclusion criteria included a prior history of virologic failure on an integrase strand transfer inhibitor (INSTI)-containing regimen, documented primary INSTI resistance and a documented HIV-1 RNA ≥50 copies/mL in the year prior to switch. Informed consent was waived due to the retrospective observational nature of the study, which utilized data collected as a part of routine clinical care.

Demographics, lab values, and clinical parameters were extracted from the charts of all eligible patients through Week 48 of treatment with B/F/TAF. Reasons for switching to B/F/TAF were obtained from a templated “ARV switch” **S**ubjective, **O**bjective, **A**ssessment and **P**lan (SOAP) note that is used by all OIC providers to document decisions and considerations surrounding ARV switch. The primary endpoint of the study was the proportion of patients with plasma HIV-1 RNA <50 copies/mL at Week 48. Secondary endpoints included the change in number of DDIs following switch to B/F/TAF, change in CD4^+^ cell count from baseline to Week 48, change in lipid parameters (total cholesterol, LDL cholesterol, HDL cholesterol, and triglycerides) from baseline to Week 48, and safety and tolerability of treatment with B/F/TAF. The change in DDIs pre- and post-switch was assessed by performing 2 DDI analyses for each patient using the University of Liverpool HIV DDI database.^[15]^ The first analysis assessed DDIs associated with the patient's preswitch ARV regimen and concomitant medications. The second analysis assessed DDIs associated with B/F/TAF and the same concomitant medications. All laboratory abnormalities and documented AEs were graded using the Division of AIDS Table for Grading the Severity of Adult and Pediatric Adverse Events.^[16]^

Descriptive statistics (frequencies, proportions, and medians with range) were calculated for participant baseline demographic and clinical characteristics, virologic outcomes, change in weight, DDIs pre- and post-switch, AEs, and discontinuations throughout the study. Descriptions of adherence were summarized based on clinician documentation in the medical record. For AEs, drug-relatedness was assigned based on whether the clinician documented a possible relationship between the AE and B/F/TAF. The Wilcoxon paired rank test was used to determine whether there were significant changes in CD4^+^ count or lipid parameters from baseline to Week 48. The Sterling Institutional Review Board (IRB) determined that the study met IRB exemption criteria based on the observational nature of the study which utilized retrospective data collected as a part of routine clinical care (Sterling IRB ID 7532). Informed consent was not utilized for this retrospective, observational cohort and was determined to not be required by the Sterling IRB.

## 3 Results

During the study period, 727 PLWH aged ≥50 years switched to B/F/TAF as a complete ARV regimen. Two-hundred and ninety-eight did not have at least 2 HIV-1 RNA measurements following switch, 53 were lost-to-follow-up immediately after switch and 245 had recently switched to B/F/TAF; of these 198 only had a single HIV-1 RNA measurement following switch and 46 did not yet have any HIV-1 RNA measurements following switch at the time of data cut-off. Seventy-nine patients had baseline HIV-1 RNA ≥50 copies/mL prior to switch hence only 350 met criteria for inclusion. The median age (range) was 57 (50, 81) years, 69 (20%) were women, and 136 (40%) were non-White (Table 1). Median number of baseline chronic comorbid conditions (range) was 5 (0, 20), and the median number of baseline concomitant medications (range) was 4 (0, 23) (Table 1). The median baseline Charlson comorbidity index score (range) was 2 (1, 8); the most common baseline comorbidities included hypertension in 203 (58%), hyperlipidemia in 179 (51%), and diabetes in 75 (21.5%). The documented median duration of HIV infection (range) was 20 (1, 40) years, and median documented number of ARV regimens prior to switch (range) was 4 (1, 11).

**Table 1:** Baseline demographic and clinical characteristics.

Characteristic	N = 350
Median age (range)	57 (50, 81)
Sex	
Male, n (%)	281 (80)
Female, n (%)	69 (20)
Race/Ethnicity	
Caucasian, n (%)	199 (57)
Black, n (%)	56 (16)
Hispanic, n (%)	80 (23)
Asian, n (%)	5 (1)
Other, n (%)	9 (3)
BMI, median (range)	27.8 (17.4, 48.3)
Weight, median (range), kg	83.9 (40.4, 157.1)
CD4^+^ cell count, median (range), cells/mm^3^	664 (58, 2327)
Co-infection	
HBV, n (%)	14 (4)
HCV, n (%)	10 (3)
Number of chronic comorbid conditions, median (range)	5 (0, 20)
Charlson comorbidity index score, median (range)	2 (1, 8)
Charlson 10-year survival percentage, median (range)	90 (0–96)
Number of baseline concomitant medications, median (range)	4 (0, 23)
Documented duration of HIV infection prior to switch, median (range), years	20 (1, 40)
Documented number of ARV regimens prior to switch, median (range)	4 (1, 11)
Documented duration of virologic suppression prior to switch, median (range), years	11 (0, 27)
Prior ARV experience	
>2 NRTIs, n (%)	288 (82)
≥1 NNRTI, n (%)	250 (71)
≥2 PIs, n (%)	93 (27)
1 INSTI, n (%)	171 (49)
>1 INSTI, n (%)	64 (18)
Regimen prior to switch	
Dual NRTI+NNRTI, n (%)	80 (23)
Dual NRTI+PI, n (n%)	45 (13)
Dual NRTI+INSTI, n (%)	193 (55)
PI+INSTI, n (n%)	8 (2)
NNRTI+INSTI, n (%)	3 (1)
Other, n (n%)	21 (6)
Rationale for switch to B/F/TAF	
Simplification, n (%)	123 (35)
DDI avoidance, n (%)	93 (27)
TDF to TAF switch	70 (20)
Comorbidities, n (%)	27 (7.5)
Side effects, n (%)	31 (9)
Other, n (%)	6 (1.5)
Historical genotypic resistance available, n (%)	103 (29)
≥1 NRTI RAM, n (%)	35 (34)
≥1 NNRTI RAM, n (%)	33 (32)
≥1 PI RAM, n (%)	37 (36)
≥1 INSTI RAM, n (%)	2 (2)^†^
Pattern of NRTI RAMs^∗^	
None, n (%)	77 (75)
M184V/I alone, n (%)	10 (10)
M184V/I+ 1 NRTI RAM, n (%)	6 (5)
M184V/I + >1 NRTI RAM, n (%)	10 (10)

ARV = antiretroviral, B/F/TAF = bictegravir/emtricitabine/tenofovir alafenamide, BMI = body mass index, DDI = drug–drug interaction, HBV = hepatitis B, HCV = hepatitis C, INSTI = integrase strand transfer inhibitor, NNRTI = non-nucleoside reverse transcriptase inhibitor, NRTI =  = nucleoside reverse transcriptase inhibitor, PI = protease inhibitor, RAMs = resistance associated mutations, TDF = tenofovir disoproxil fumarate.

∗ Total with available historical genotypes used as denominator.

† Two patients with minor INSTI RAMs.

The most common regimen prior to switch consisted of an INSTI plus two nucleoside reverse transcriptase inhibitors (NRTIs) in 193 (55%); 80 (23%) were switched from regimens containing a non-nucleoside reverse transcriptase inhibitor (NNRTI) plus 2 NRTIs; 45 (13%) were switched from a PI plus 2 NRTIs, and 32 (9%) were switched from non-traditional ARV regimens consisting of either a PI plus an INSTI, an NNRTI plus an INSTI, or an ARV regimen consisting of ≥3 ARV classes (Table 1). Notably, 88/350 (25%) were switched from tenofovir disoproxil fumarate (TDF)-containing regimens. The most common documented reason for switch was simplification in 35% followed by avoidance of DDIs in 27% (Table 1).

Historical genotypic resistance tests were available for 103 (29%) patients, of whom 35 (34%) had NRTI resistance, 33 (32%) had NNRTI resistance, 37 (36%) had PI resistance, and 2 (2%) had INSTI resistance (both with minor INSTI resistance associated mutations [RAMs] not associated with resistance to bictegravir). Twenty-six (25%) patients had an M184 V/I mutation present on historical resistance testing: 6 (5%) had an M184 V/I plus 1 additional NRTI RAM and 10 (10%) had an M184 V/I plus at least 2 additional NRTI RAMs (Table 1). At baseline, the median total cholesterol was 183.5 mg/dL, median LDL cholesterol was 106 mg/dL, median HDL cholesterol was 46 mg/dL, and median triglycerides were 139 mg/dL.

**Figure 1:**
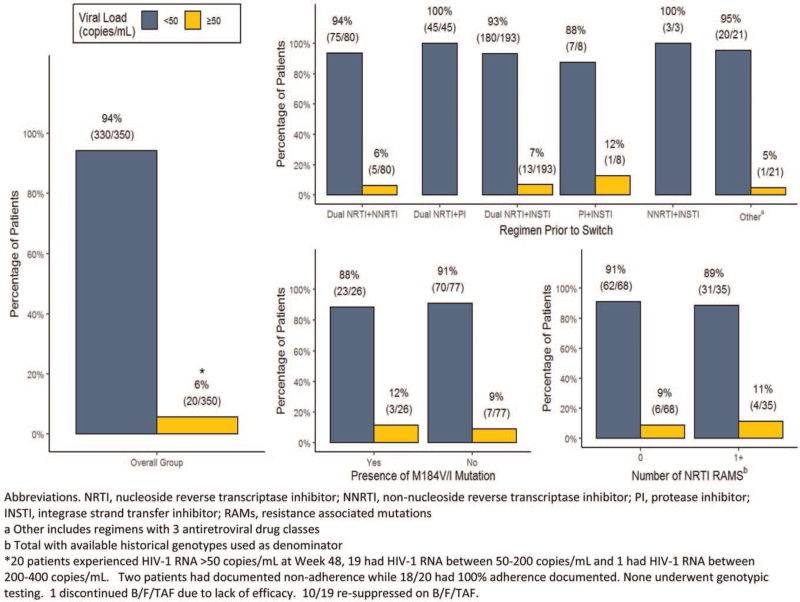
Subgroup analysis of virologic outcomes at Week 48.

At Week 48, 330/350 (94%) patients maintained virologic suppression, while 20/350 (6%) had an HIV-1 RNA ≥50 copies/mL (Fig. 1). Nineteen of these patients had an HIV-1 RNA between 50 and 200 copies/mL, and 1 patient had an HIV-1 RNA between 200 and 400 copies/mL. Among those with HIV-1 RNA ≥50 copies/mL, 2 had documented non-adherence, while 18/20 had 100% adherence documented. Nine of these patients had historical genotypes available. None had INSTI mutations and 6 had no NRTI mutations. One had an M184 V plus 1 thymidine analog mutation (TAM), 1 had an M184 V plus 2 TAMs, and 1 had a single TAM without an M184 V/I. None of the non-suppressed patients underwent post-switch genotypic testing. Only one discontinued B/F/TAF due to lack of efficacy; this patient had an HIV-1 RNA of 190 copies/mL at the time of discontinuation. Historical genotypic testing demonstrated an M184 V plus 1 TAM without INSTI mutations. The patient was subsequently switched to DTG plus darunavir/c and achieved an HIV-1 RNA of <50 copies/mL 12 weeks after the switch. Ten of 19 patients resuppressed on B/F/TAF after the study period ended.

Subgroup analyses revealed no difference in virologic response at Week 48 based on the presence of historical NRTI resistance; 88% of those with an M184 V/I maintained an HIV-1 RNA <50 copies/mL versus 91% of those without an M184 V/I. Eighty-nine percent of patients with at least 1 NRTI RAM maintained virologic suppression at Week 48 versus 91% with no NRTI RAMs (Fig. 1). The 2 patients with minor INSTI RAMs on historical genotype both maintained an HIV-1 RNA <50 copies/mL at Week 48. There was also no difference in response based on regimen prior to switch, with high response rates observed among those switched from regimens consisting of 2 NRTIs plus a third agent and non-traditional ARV regimens (Fig. 1). There was no significant change in median CD4^+^ count from baseline to Week 48 (+7 cells/mm^3^, 95% confidence interval [CI]: –9; 29).

Prior to switching to B/F/TAF, a total of 140 potential DDIs were identified in 121/350 (35%) patients taking a boosting agent or rilpivirine at baseline (Table 2). The most common included interactions between boosting agents and statins in 81 patients (23%) and boosting agents and phosphodiesterase type 5 inhibitors in 25 patients (7%) (Table 2). In all cases, DDIs were mitigated with the switch to B/F/TAF.

**Table 2:** Avoidance of drug–drug interactions (DDIs) following switch to B/F/TAF.

Baseline ARV	Concomitant medication	DDI resolution following switch to B/F/TAF N (%) total n = 350
Ritonavir or cobicistat containing regimen	Statins	81 (23)
Ritonavir or cobicistat containing regimen	PDE5 inhibitors	25 (7)
Ritonavir or cobicistat containing regimen	Factor Xa inhibitors	3 (1)
Ritonavir or cobicistat containing regimen	P2Y12 inhibitors	4 (1)
Ritonavir or cobicistat containing regimen	Warfarin	1 (0.3)
Ritonavir or cobicistat containing regimen	Inhaled or intranasal steroids	16 (5)
Ritonavir or cobicistat containing regimen	HCV NS3/4A protease inhibitor	1 (0.3)
Rilpivirine	PPIs	6 (2)
Rilpivirine	H2 blockers	3 (1)

ARV = antiretroviral, B/F/TAF = bictegravir/emtricitabine/tenofovir alafenamide, H2 = histamine type 2, PDE5 = phosphodiesterase type 5, PPI = proton pump inhibitor.

**Figure 2:**
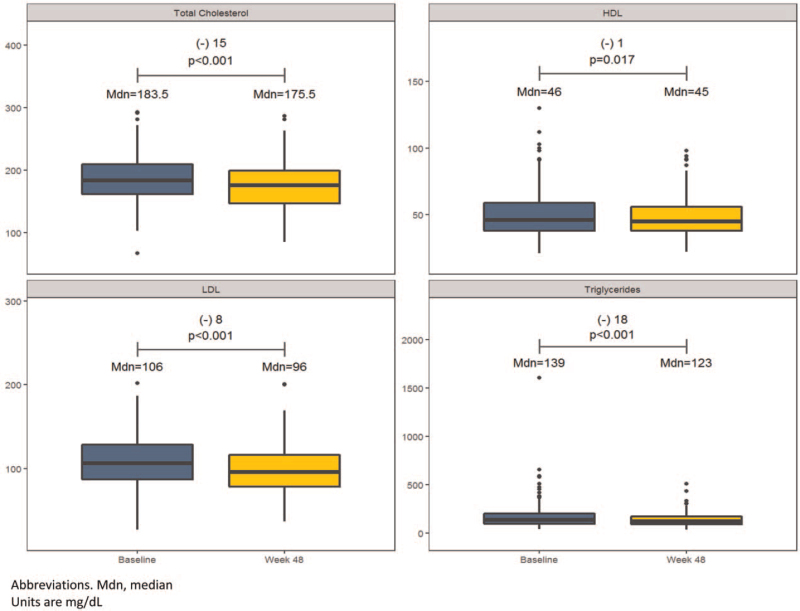
Changes in lipid parameters through Week 48.

There were significant changes in all lipid parameters from baseline to Week 48. Median total cholesterol decreased by 15 mg/dL (95% CI: –21.5; –9.5), median HDL cholesterol decreased by 1 mg/dL (95% CI: –3.5; –0.5), median LDL cholesterol decreased by 8 mg/dL (95% CI: –15.5; –5.5), and median triglycerides decreased by 18 mg/dL (95% CI: –31; –10.5) (Fig. 2). At baseline 179 (51%) patients were on lipid-lowering therapy; during the study period 42 (12%) initiated lipid-lowering therapy and 11 (3%) discontinued lipid-lowering therapy.

Drug-related AEs occurred in 51 patients (15%) throughout the study period; the most common were fatigue in 14 (4%), weight gain in 11 (3%), and arthralgia in 11 (3%) (Table 3). Most drug-related AEs were Grade 1 in severity, with only 16 patients (5%) experiencing Grade 2–5 drug-related AEs (Table 3). Eight patients experienced drug-related AEs that led to B/F/TAF discontinuation; 5 had an available HIV-1 RNA measurement at B/F/TAF discontinuation, and all were virologically suppressed. There were no serious adverse events or deaths during the study period. Grade 3–4 lab abnormalities occurred in 25 (7%) patients and were not responsible for any B/F/TAF discontinuations (Table 3).

**Table 3:** Safety and tolerability.

Characteristic	B/F/TAF (N = 350) N (%)
Drug-related adverse events (AEs)^∗^	51 (15)
Grade 2–5 drug-related AEs	16 (5)
Leading to B/F/TAF discontinuation^†^	8 (2)
Grade 3–4 lab abnormalities^‡^	25 (7)
Serious AEs	0
Death	0

∗ The most common drug-related AEs were fatigue (4%), weight gain (3%), and arthralgia (3%).

† These included diarrhea (2), dizziness (2), arthralgia (2), creatinine elevation (1) and abdominal pain (1).

‡ These included hypertriglyceridemia (14), hyperglycemia (9), hypercholesterolemia (1), and transaminitis (1).

Median increase in weight through 48 weeks was 1.04 kg (95% CI: 0.63, 1.44); this corresponded to a median percent change from baseline in weight of +1.2% (95% CI: 0.8%, 1.7%). Throughout the study period, 123 (37%) patients experienced weight loss, 13 (4%) experienced no change in weight, and 196 (59%) experienced weight gain. Sixty-three (19%) experienced ≥5% weight gain and 15 (5%) experienced ≥10% weight gain, whereas 23 (7%) experienced ≥5% weight loss and 6 (2%) experienced ≥10% weight loss.

## 4 Discussion

In our cohort switching to B/F/TAF was associated with maintenance of virologic suppression in 94% of patients aged ≥50 years at Week 48, the majority of whom had multiple comorbid conditions requiring concomitant medications. Among the 6% not suppressed at Week 48, no post-switch genotypes were obtained as all had low-level viremia with an HIV-1 RNA between 50 and 400 copies/mL. Approximately half subsequently went on to achieve virologic suppression on B/F/TAF after the study period ended, and only one patient discontinued due to lack of efficacy. Virologic outcomes did not differ significantly by regimen prior to switch or the presence of historical NRTI resistance (Fig. 1). These data are consistent with results from clinical trials and the multicountry BICStaR cohort, which revealed suppression rates of 91% to 93% in older PLWH who were switched to B/F/TAF through Week 48.^[11–13]^ Study 380–4449 reported durable virologic efficacy with 94% of PLWH aged ≥65 years maintaining an HIV-1 RNA <50 copies/mL after switching to B/F/TAF at Week 72.^[10]^ Many in these prior studies were switched to B/F/TAF from regimens consisting of 2 NRTIs plus a third agent. Our study also included a small proportion switched from non-traditional regimens not evaluated in prior studies, including 2-drug INSTI-based regimens and regimens containing ≥3 ARV classes, and revealed similarly high suppression rates among this subgroup (Fig. 1).

A recent analysis of study participants with archived resistance switched to B/F/TAF from PI- or DTG-based regimens revealed that 97.9% of participants with pre-existing NRTI or INSTI resistance and 96% with an archived M184 V/I maintained virologic suppression at Week 48.^[17]^ Similar results were observed in study 380–4030, which evaluated the efficacy of switching virologically suppressed adults on DTG plus either F/TAF or F/TDF to B/F/TAF. Among 565 participants enrolled in this study, 24% were found to have pre-existing NRTI resistance, 4% had pre-existing INSTI resistance, and 14% had an M184 V/I at baseline.^[18]^ At Week 48, 100% of participants with NRTI or INSTI resistance and pre-existing M184 V/I switched to B/F/TAF maintained suppression.^[18]^ In the BRAAVE study, pre-existing resistance among virologically suppressed African Americans switched to B/F/TAF was also found to have no significant impact on virologic outcomes, with 100% of participants with baseline NRTI or INSTI resistance, and 100% with M184 V/I maintaining virologic suppression at Week 48.^[19]^ Among these studies, no participant developed treatment-emergent resistance.

Similar findings have also been demonstrated outside of the clinical trial setting, with 8/8 patients in the BICStaR cohort with pre-existing M184 V/I achieving virologic suppression on B/F/TAF at Month 12.^[13]^ Data from a larger real-world cohort of 33 treatment-experienced patients with documented M184 V/I mutations who switched to B/F/TAF demonstrated that 30/33 achieved an HIV-1 RNA <200 copies/mL at Month 12.^[20]^ Our results add to data demonstrating the efficacy of B/F/TAF in those with pre-existing NRTI resistance, including those with an M184 V/I mutation. We observed high suppression rates of 89% in patients with pre-existing NRTI resistance and 88% in patients with historical M184 V/I at Week 48 (Fig. 1). Two patients with minor INSTI RAMs at baseline both maintained virologic suppression at Week 48. Overall, these data reinforce the efficacy of B/F/TAF in treatment-experienced patients with pre-existing resistance. This is particularly relevant for older PLWH, as this group is more likely to have treatment-limiting ARV resistance compared with younger populations.^[21]^

In our study switching to B/F/TAF was associated with mitigation of DDIs in 35% of patients, all of whom were on a boosting agent or rilpivirine at baseline (Table 2). Other studies examining the impact of switching to B/F/TAF on DDIs have revealed similar findings. A multicenter, retrospective cohort study of 411 treatment-experienced PLWH on at least 1 concomitant medication reported a decrease in the number of total DDIs from 552 on the baseline regimen to 188 after switching to B/F/TAF.^[22]^ A significant reduction in DDI score (higher scores being indicative of more severe interactions) was also observed after switching to B/F/TAF in patients receiving concomitant medications for a variety of comorbidities including cardiovascular disease, neurological/psychiatric disorders, chronic pain, inflammation, gastrointestinal/urologic conditions, and conditions requiring hormonal therapy.^[22]^ Many have warned about the complications associated with DDIs in older PLWH as comorbidities accumulate and have found that polypharmacy and DDIs are a source of increased morbidity and higher healthcare costs in this population.^[23–26]^ A recent analysis of AEs due to inappropriate prescribing in older PLWH demonstrated that 30% of PLWH aged ≥65 years experienced ≥1 AE due to inappropriate prescribing, and the risk of having an AE increased as the total number of non-HIV medications increased (adjusted odds ratio 1.2, 95%CI: 1.1–1.3).^[23]^ Such studies underscore the importance of identifying ARVs with favorable DDI profiles for use in older PLWH. Our data support B/F/TAF as a preferred option to minimize DDIs in this population.

Switching to B/F/TAF resulted in significant reductions in total cholesterol, LDL cholesterol, and triglycerides in our cohort (Fig. 2). In other clinical studies switching to B/F/TAF has been associated with variable changes in lipid parameters. Switching from regimens containing boosted PIs was associated with significant declines in triglycerides and total cholesterol to HDL ratio through Week 48,^[27]^ and small numerical declines in total cholesterol, LDL cholesterol, triglycerides, and total cholesterol to HDL ratio among those continuing B/F/TAF through Week 96.^[8]^ In comparison, switching from DTG/ABC/3TC was not associated with any significant changes in lipid parameters through Week 48,^[28]^ and small numeric increases in LDL cholesterol were observed among those continuing B/F/TAF through Week 96.^[7]^ In studies focused on older PLWH, switching to B/F/TAF in virologically suppressed adults aged ≥65 years was associated with significant declines in total cholesterol and triglycerides; however, 93% of patients in this study were switched from regimens containing cobicistat.^[11]^ These data are consistent with our results, which additionally demonstrated a significant decline in LDL cholesterol following switch to B/F/TAF. However, only 54% of patients in our cohort were switched from regimens containing cobicistat or ritonavir, and 25% were switched from regimens containing TDF. These results are somewhat unusual given the known lipid-lowering effects of TDF compared with TAF^[29–31]^ and suggest that B/F/TAF may be more “lipid-neutral” compared with other ARV regimens. Given the increased risk of vascular disease and metabolic complications among older PLWH,^[4]^ these data are critical and highlight B/F/TAF as a potentially “lipid-friendly” ARV option for this population.

Safety data from our cohort demonstrated that B/F/TAF was well-tolerated with Grade 2–5 drug-related AEs and discontinuations due to AEs occurring in only 5% and 2% of older PLWH, respectively (Table 3). There were no serious drug-related AEs or deaths. At Week 48, median percent increase in weight from baseline was 1.2%; this corresponded to an absolute median increase in weight of 1.04 kg. These results are consistent with safety analyses from other trials evaluating B/F/TAF in older adults and highlight its favorable tolerability and safety profile in this group.^[10,12]^

Limitations of our study include the retrospective nature of the analysis, the inability to control for confounding factors and the fact that data are from a single center in the Southeastern United States, which limits generalizability to other populations. We also acknowledge that a significant proportion of PLWH aged ≥50 years switched to B/F/TAF in our cohort were not eligible for study inclusion due to lost to follow-up immediately following switch (53/727) and not accruing enough follow-up time to have at least 2 HIV-1 RNA measurements following switch (245/727). The lack of inclusion of these populations may represent a source of selection bias as the strategy of only including those switched to B/F/TAF with at least 48 weeks of follow-up data may favor the switch regimen. However, this is the first real-world study evaluating the efficacy and safety of switching to B/F/TAF in a racially diverse cohort of older PLWH from the United States and provides important insight on the potential benefits of this treatment option in our rapidly growing ageing population.

In conclusion, switching to B/F/TAF was associated with high virologic suppression, improvement in lipid parameters, and avoidance of DDIs in a large proportion from this real-world cohort of older PLWH. These data support B/F/TAF as a potential switch option in PLWH aged ≥50 years with no history of virologic failure on an INSTI-based regimen and highlight some important benefits of use to consider in this population.

## Acknowledgments

All authors listed have contributed sufficiently to the project to be included as authors, and all those who are qualified to be authors are listed in the author byline. Each author's specific contribution is noted below.

## Author contributions

Charlotte-Paige Rolle – Gathered, analyzed, and interpreted the data necessary for the writing of this article, drafted and heavily revised the paper, provided the final review for the approval of submission, and has agreed to be fully accountable of the work ensuring its accuracy and integrity.

Vu Nguyen – Responsible for the conception and design of the study, assisted in revision of the article, provided the final review for the approval of submission, and has agreed to be fully accountable of the work ensuring its accuracy and integrity.

Kiran Patel – Responsible for the conception and design of the study, assisted in revision of the article, provided the final review for the approval of submission, and has agreed to be fully accountable of the work ensuring its accuracy and integrity.

Dan Cruz – Responsible for the conception and design of the study, assisted in revision of the article, provided the final review for the approval of submission, and has agreed to be fully accountable of the work ensuring its accuracy and integrity.

Edwin DeJesus – Responsible for the conception and design of the study, assisted in revision of the article, provided the final review for the approval of submission, and has agreed to be fully accountable of the work ensuring its accuracy and integrity.

Federico Hinestrosa – Responsible for the conception and design of the study, assisted in revision of the article, provided the final review for the approval of submission, and has agreed to be fully accountable of the work ensuring its accuracy and integrity.

**Conceptualization:** Charlotte-Paige Rolle, Kiran Patel, Edwin DeJesus.

**Data curation:** Charlotte-Paige Rolle, Dan Cruz, Edwin DeJesus, Federico Hinestrosa.

**Formal analysis:** Charlotte-Paige Rolle, Vu Nguyen.

**Funding acquisition:** Charlotte-Paige Rolle, Kiran Patel.

**Investigation:** Charlotte-Paige Rolle, Kiran Patel.

**Methodology:** Charlotte-Paige Rolle.

**Project administration:** Charlotte-Paige Rolle.

**Resources:** Charlotte-Paige Rolle.

**Supervision:** Charlotte-Paige Rolle, Edwin DeJesus.

**Validation:** Charlotte-Paige Rolle.

**Visualization:** Charlotte-Paige Rolle.

**Writing – original draft:** Charlotte-Paige Rolle.

**Writing – review & editing:** Charlotte-Paige Rolle, Kiran Patel, Dan Cruz, Edwin DeJesus, Federico Hinestrosa.
